# Weakening of Indian Summer Monsoon Rainfall due to Changes in Land Use Land Cover

**DOI:** 10.1038/srep32177

**Published:** 2016-08-24

**Authors:** Supantha Paul, Subimal Ghosh, Robert Oglesby, Amey Pathak, Anita Chandrasekharan, RAAJ Ramsankaran

**Affiliations:** 1 Interdisciplinary Program in Climate Studies, Indian Institute of Technology Bombay, Mumbai- 400076, India; 2 Department of Civil Engineering, Indian Institute of Technology Bombay, Mumbai- 400076, India; 3 Department of Earth & Atmospheric Sciences, University of Nebraska, Lincoln, NE, USA; 4 School of Natural Resources, University of Nebraska, Lincoln, NE, USA

## Abstract

Weakening of Indian summer monsoon rainfall (ISMR) is traditionally linked with large-scale perturbations and circulations. However, the impacts of local changes in land use and land cover (LULC) on ISMR have yet to be explored. Here, we analyzed this topic using the regional Weather Research and Forecasting model with European Center for Medium range Weather Forecast (ECMWF) reanalysis data for the years 2000–2010 as a boundary condition and with LULC data from 1987 and 2005. The differences in LULC between 1987 and 2005 showed deforestation with conversion of forest land to crop land, though the magnitude of such conversion is uncertain because of the coarse resolution of satellite images and use of differential sources and methods for data extraction. We performed a sensitivity analysis to understand the impacts of large-scale deforestation in India on monsoon precipitation and found such impacts are similar to the observed changes in terms of spatial patterns and magnitude. We found that deforestation results in weakening of the ISMR because of the decrease in evapotranspiration and subsequent decrease in the recycled component of precipitation.

Indian summer monsoon rainfall (ISMR) contributes 80% of the total annual precipitation in India^1^,^2^ and controls the agricultural productivity and economy of the country. The variability of ISMR at different time scales, from intra-seasonal to multi-decadal, impacts the life of more than one billion people^2^,^3^. ISMR must be reliably simulated and projected for sustainable water resources management, agricultural planning towards adaptation to climate change^4^,^5^.

Variabilities in ISMR at multiple space-time scales, from intra-seasonal to multi-decadal and from subdivisional to regional, affect agricultural productivity in the country and hence the gross domestic product (GDP)^3^,^4^,^5^. Observations indicate that ISMR shows a statistically significant decreasing trend since 1950^6^. Multiple hypotheses have been proposed to explain this decrease. *Bollasina et al.*^7^ concluded that Northern India had higher aerosol emissions, resulting in cooling over the land mass. This factor weakened the temperature gradient between the Northern and Southern hemispheres, thereby decreasing monsoon rainfall in Central North East India. Another hypothesis proposed by *Rao et al.*^8^,^9^ considers warming of the Southern Indian Ocean (SIO) as the key driver for the decrease in ISMR. Warming of the SIO weakens the meridional Sea Surface Temperature (SST) gradient (ΔSST), which in turn weakens the meridional Hadley circulation, thereby reducing ISMR^10^. In a recent study, *Roxy et al.*^11^ found that changes in ISMR may be attributed to a warming of Western Indian Ocean and a subsequent weakening of the temperature gradient in SST over the Indian Ocean. Interestingly, all of these hypotheses are supported by different models, but none of the models considers the recent changes in LULC over India. Land surface feedback to the atmosphere plays a major role in climate systems, as reported in various studies^12^,^13^,^14^,^15^,^16^*. Niyogi et al.*^17^ indicated possible reductions to the Indian monsoon from changes in land surface. They speculated that the agricultural intensification in North India during a pre-monsoon period altered the regional monsoon circulation. Precipitation resulting from local land surface Evapotranspiration (ET) is known as recycled precipitation. *Pathak et al.*^18^ found that evapotranspiration from land surface vegetation plays a major role during the end of a monsoon. They observed that, during the initial phase of a monsoon, oceanic sources play a major role, and the soil is recharged with moisture. However, during the latter half of a summer monsoon (August and September), land surface ET increases as recycled precipitation increases, a pattern that is more prominent in the Ganga Basin and Northeast India. This recycled precipitation accounts for approximately 20–25% of the rainfall in North India (Ganga Basin) and Northeast India during August and September^18^. Hence, deforestation associated with changes in LULC may affect ET and may significantly affect monsoon rainfall. *Ozturk et al.*^19^ found that changes in ET may affect drought intensity. These results agree with a model-based study by *Devaraju et al.*^20^, in which modeling of deforestation across the global monsoon region showed an 18% decline in precipitation in India. However, these models consider hypothetical scenarios for full deforestation across the entire monsoon region; therefore, they cannot quantify the impacts of the recent LULC changes that have taken place in India. Regional modeling also reveals the impacts of deforestation on rainfall in Peninsular India and Srilanka^21^. There is a compelling need to understand the impacts of recent LULC changes in India on monsoon rainfall. Here, we attempt to perform a sensitivity analysis using regional model simulations using weather research and forecasting (WRF), which considers large-scale deforestation in recent decades. We consider the LULC from the 1980s (LULC map of 1987) and the 2000s (LULC map of 2005) [details in Methods] in two separate runs [details in Methods], each for 2000–2010. As the LULC are obtained for these two time scales using different methods from different sources [details in Methods], the detected deforestation may have considerable uncertainty. Here, we present these regional runs as a representative experiment to understand the impacts of large-scale deforestation on monsoon rainfall. The differences in simulated precipitation between the LULCs of 1987 and 2005 provide an estimate of changes in precipitation due to large-scale deforestation in India.

## Results

### Changes in LULC

**Figure 1:**
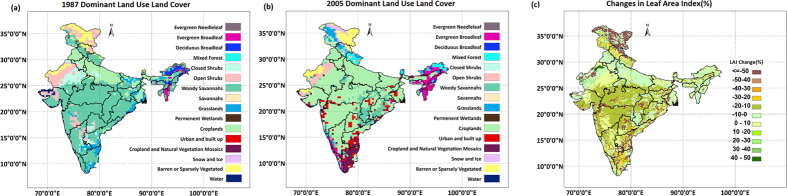
Land Use and Land Cover (LULC) changes in India in recent decades. (**a**) LULC map for 1987 (**b**) and 2005 (**c**) Changes (%) in Leaf Area Index in 2005 from 1987. Maps are prepared with ArcGIS10.0 (http://www.esri.com/software/arcgis/arcgis-for-desktop). The LULC from 1987 is obtained from AVHRR, and from 2005 is obtained from MODIS.

During the 1980s, the dominant LULC type in India was woody savanna, which was mostly forest land, and this type was especially dominant over Central India, the majority of Peninsular India and Northeast India (Fig. 1). Because of development and agricultural intensification, these regions were largely deforested, and woody savanna was converted to cropland. LULC data from 2005 show that cropland is the dominant LULC type in the country, and it is important to understand the effect of this change on monsoon precipitation. For example, in Northeast India, the dominant LULC changed from woody savanna to evergreen broadleaf because of increased tea plantations. In general, approximately 20% of leaf area index (LAI) has been reduced in the core monsoon zone (Fig. 1(c)). To understand the effects of such changes, we simulated the regional monsoon with WRF coupled (details in supplementary) with Community Land Model (CLM4.0), forced with different LULC types. However, significant uncertainty resides in the estimation of LULC changes, specifically because of the following (details in Methods):

1. The historical satellite images are of coarse resolution.
2. The LULC from the two periods were obtained from two different sources.
3. Differences exist in the classification schemes and methods between the data extraction procedures for the two periods.

### Evaluation of WRF

First, we evaluated WRF simulations for 2000–2010 while imposing 2005 LULC and large-scale forcing provided by ECMWF (ERA-Interim) as boundary conditions [details in Methods]. Details of the LULC datasets used and their application in the WRF model, along with complete information on the model setup, model resolution and rainfall observations used are given in the section ‘Methods’.

**Figure 2:**
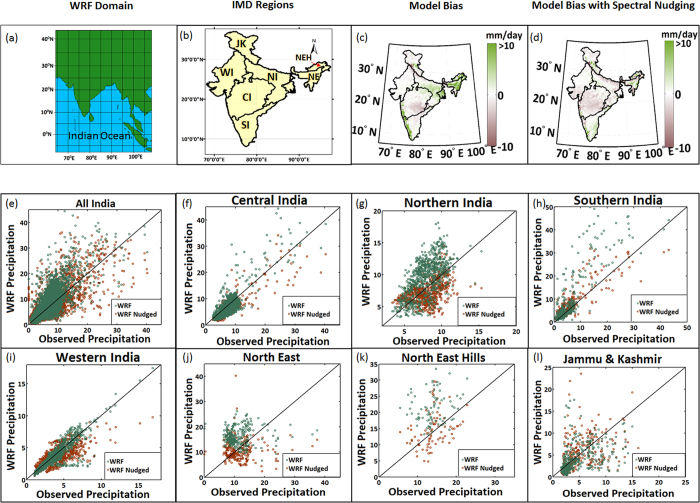
Evaluation of regional simulations by Weather Research and Forecasting (WRF) model. (**a**) The domain considered for WRF simulations, (**b**) IMD meteorologically homogeneous regions, (**c**) Bias in regional model simulation without nudging and (**d**) with spectral nudging. (**e–l**) The scatter plots between WRF simulations (with and without nudging) and observed precipitation for all India and its different homogeneous regions (**e–l**). The period considered here is 2000–2010, and the LULC considered is for 2005. Maps are prepared with MATLAB R2012b (http://in.mathworks.com/products/new_products/release2012b.html). The observed precipitation data are obtained from the India Meteorological Department.

Figure 2(a) presents the domain used for the study. We considered a slightly larger domain than the Indian subcontinent to ensure that specific monsoon features were properly incorporated, specifically those associated with the variabilities of SST in the Indian Ocean and temperature over the Tibetan plateau. The meteorologically homogeneous regions as mentioned by India Meteorological Department (IMD) are presented in Fig. 2(b).

We performed regional simulations for 2000–2010 with and without spectral nudging to understand whether nudging could provide a better simulation of monsoon rainfall. Nudging is not performed at surface level (below planetary boundary layer); hence, the low-level variables are not forced to match with those from reanalysis. We compared the results with the observed gridded precipitation data from India Meteorological Department (IMD) [details in Methods]. Bias in simulated precipitation was reduced when the model was nudged spectrally (Fig. 2(c,d)), specifically in regions of high precipitation such as Northeast India and the West Coast region. This pattern was also evident from the scatter plots for all of India as well for its homogeneous regions (Fig. 2(e–l)). Each point in the scatter plot represents a grid point with its mean observed or simulated precipitation during the summer monsoon from 2000 to 2010. North and Northeast India showed better agreement. Spatially averaged seasonal precipitations (JJAS) for all of India and its homogeneous regions, as simulated by WRF with nudging, have higher correlations with observed JJAS than WRF without nudging, except in Jammu and Kashmir (Supplementary Fig. S1). This finding also indicates that WRF with nudging improves the simulation of interannual variation of monsoon precipitation. We also evaluated the regional runs in terms of spatial variability, represented by Probability Density Functions (PDF). PDFs show better agreement for nudged simulations with respect to the free runs when evaluated with observed data in the entire country as well as for different regions, specifically North India, Western India, and the northeastern hills (Supplementary Fig. S2).

### Impacts of Changes in LULC on Monsoon Rainfall

**Figure 3:**
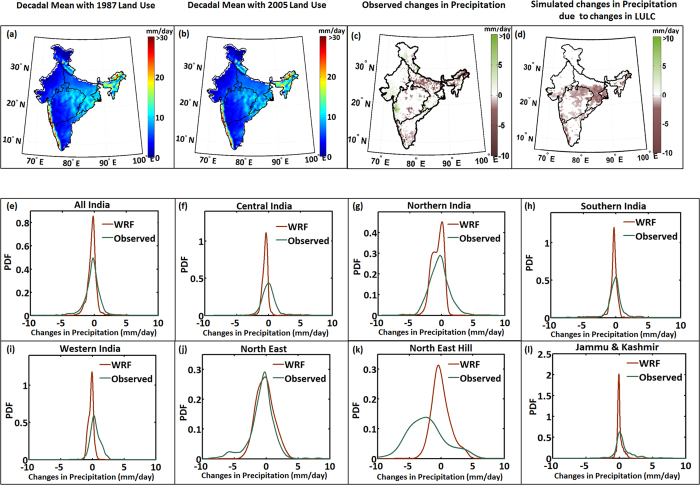
Changes in Monsoon Precipitation due to changes in LULC and their consistency with the declining patterns of ISMR. Simulated average monsoon precipitation with (**a**) 1987 and (**b**) 2005 LULC and (**d**) their differences, which are consistent with observed changes in ISMR (**c**) from the 1980s to the 2000s. PDFs representing the spatial variability of changes or differences with different LULC are presented for all India and its different homogeneous regions (**e–l**). Maps are prepared with MATLAB R2012b (http://in.mathworks.com/products/new_products/release2012b.html).

We performed WRF-CLM simulations with nudging and imposed LULC for both the 1980s (1987) and the 2000s (2005). The duration of the run was 2000–2010. The mean monsoon rainfall (JJAS) obtained with both the LULCs is presented in Fig. 3(a,b), with their differences shown in Fig. 3(d). The differences are negative when the 1980s LULC run is subtracted from the 2000s LULC run. Negative differences denote the changes in monsoon rainfall due to large-scale conversion of woody savannah to crop land as obtained from remote sensing images from the 1980s and the 2000s. These negative differences are larger and more prevalent in Northeast India and the Ganga Basin, where *Pathak et al.*^18^ found higher contributions of recycled precipitation to monsoons. We also plotted the observed difference in monsoon rainfall between the 2000s and the 1980s (Fig. 3(c)). The observed data show a strong decline in monsoon rainfall in Northeast India, the Ganga Basin and some regions of Central India. Hence, similarities are observed between the observed changes and the differences obtained from the LULC runs. This observation indicates that the effects of an experimental large-scale LULC change in India—specifically, the change from forest land to crop land—may lead to a significant decline in ISMR, similar in extent to the observed changes. We further plotted the PDF representing the spatial variability of observed changes and WRF simulated changes in India as well as its regions due to deforestation (Fig. 3(e–l)). The similarities in the PDFs showing changes between the “observed” and “simulated LULC effects” for Northeast India may indicate that the role of deforestation on precipitation will be similar to observed changes in the high-recycling precipitation zone. For all regions, except the northeastern hills and Central India, the PDFs agreed closely. We also tested the statistical significance of the similarities between the PDFs. It is very unlikely that the changes in observed and simulated precipitation (due to LULC change) will follow exactly the same distribution; hence, the Kolmogorov-Smirnov test fails. We also performed a t-test to determine whether both of the changes have a similar mean and standard deviation. We found that the similarities in changes between the “observed” and “simulated LULC effects” are statistically significant for Northeast India, South India and North India. The significance level is 0.05. This further strengthens our hypotheses that changes in LULC play a major role in the changing patterns of Indian monsoons and that the impacts of large-scale deforestation are similar in extent to the observed recent decline in monsoons. Poor agreement in the northeastern hills can be attributed to: first, the poor quality of gridded data because the region lacks stations, and second, the model is unable to produce the fine-resolution precipitation required for hilly regions. The resolution considered here is 36 km, which is not sufficient to simulate the spatial variability of precipitation in mountainous regions. However, the simulated differences do not explain the changes in monsoon precipitation fully, and significant uncertainty is associated with the detected LULC changes. The unexplained parts of the monsoon decline are due to changes in SST, aerosol emissions and other large-scale circulations.

We also computed monthly changes or differences in precipitation during monsoon months separately (Supplementary Fig. S3). The observed monthly changes in precipitation are not uniform across months (Supplementary Fig. S3(a–d)) and show greater decline during August, when the amount of recycled precipitation is higher^18^. The differences in rainfall due to changes in LULC are greatest during August and September, with the WRF simulations when ERA-interim reanalysis (from ECMWF) is used. We also test our hypothesis with WRF simulations forced with CFSR reanalysis and find decline in monsoon precipitation due to LULC changes (Supplementary Fig. S3(i–l)).

**Figure 4:**
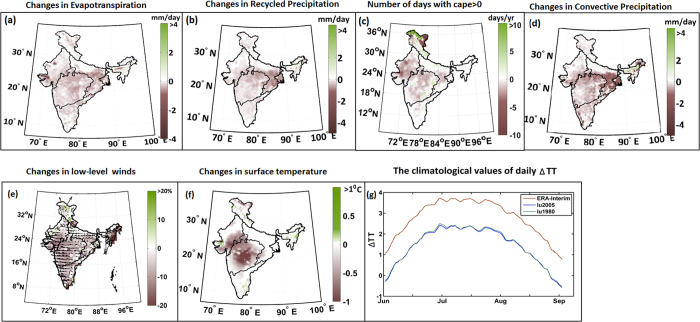
Differences in (**a**) ET, (**b**) recycled precipitation, (**c**) number of days with CAPE>0, and (**d**) convective precipitation between the regional simulations with 1987 and 2005 LULC, during the period 2000–10. Maps are prepared with MATLAB R2012b (http://in.mathworks.com/products/new_products/release2012b.html).

We further investigated the mechanism through which the changes in LULC affected monsoon precipitation. We found decrease in ET (Fig. 4(a)) due to deforestation and conversion of woody savanna to crop land. Compared to cropland, woody savanna has higher root depth; hence, it also has higher water intake resulting in higher ET. To understand the impacts of ET, we applied Dynamic Recycling Model (DRM)^22^ to quantify the amount of precipitation ET generated. DRM is applied to both the WRF simulations with 1980s and 2005 LULC. The differences in recycled precipitation as obtained from the two simulations (2005 LULC- 1980 LULC) are presented in Fig. 4(b), and the results are consistent with those of ET, as expected. The results show a decrease in recycled precipitation due to a decrease in ET. We also found that this decrease is higher at the Ganga Basin and Northeast India as expected, where the observed recycling ratio (ratio of recycled to total precipitation) is also high^18^. This higher decrease of recycled precipitation is consistent with the overall decline of the Indian monsoon. We also computed the convective available potential energy (CAPE) from both the simulations and found a very modest decrease in the number of days with CAPE>0 (Fig. 4(c)). A decrease in convective precipitation suggests an increase in atmospheric stability, a finding that is consistent with reduced total precipitation. Low CAPE is presumably due to a reduced supply of moisture, which is due to a decline in ET because of the LULC changes. The decline of ET and recycled precipitation is larger during the end of a monsoon (Supplementary Fig. S4), and this is consistent with overall declines in precipitation during August and September. We also present the simulated differences in wind fields and surface temperature (Fig. 4(e,f)). We find that impact of changes in LULC over surface temperature is prominent in Central and some part of Northern India (Fig. 4(f)). The simulated changes in wind field show a slight weakening of both south westerlies from the Arabian Sea to central India and winds from the Bay of Bengal to the Ganga Basin. Such changes result into lower moisture fluxes from moisture sources to sinks with weakening monsoon precipitation. However, the observed changes in wind fields as obtained from ERA-interim (Supplementary Fig. S5) shows a strengthening of westerlies from the Arabian Sea to west-central India but a declining of the same to South India. The observed data show the weakening of the wind from the Bay of Bengal to North India and the Ganga Basin. The simulated temperature remains unaltered over the Ganga Basin and Northeast India with the changes in LULC, where maximum changes in recycled precipitation are simulated. We also compute the tropospheric temperature gradient (∆TT) to understand the synoptic scale processes. The tropospheric temperature (TT) is defined as the average of temperature vertically between 200 hPa and 700 hPa. ∆TT is defined as the difference between TT spatially averaged over two boxes: 64°E-108° E, 10°N-35°N and 64°E-108° E, 7.8°S-10°N. The extent of the boxes is limited by the extent of WRF domain. No significant changes (Fig. 4(g)) are observed in simulated ∆TT due to LULC changes. This is expected and consistent with our conclusion, suggesting that changes in LULC lead to a decrease in recycled precipitation; however, the large-scale circulation affecting moisture transport from distant oceanic moisture sources remains the same. ∆TT is a proxy for large-scale circulation, which does not change when LULC changes. We also plotted the ∆TT from ERA-interim reanalysis dataset. Significant differences are observed between the simulated and reanalyzed ∆TT (Fig. 4(g)). In spectral nudging^23^,^24^, the nudging term is spectrally expanded in both the zonal and meridional directions over the entire domain for the waves under selected wave numbers (in our case 3). All the other waves are filtered out. The deviation of ∆TT as simulated from reanalysis probably originates from the selection of the threshold wave number. Further decrease in the threshold wave number would probably result in merging simulated ∆TT to the reanalysis.

### Discussions and Conclusion

Here, we performed a sensitivity analysis to quantify the impacts of large-scale conversion from woody savannah to crop land in India on monsoon precipitation. We found such a change results in decreased ET and subsequently a decrease in recycled precipitation leading to a decline in monsoon precipitation. This decline is similar in extent to the observed recent decadal weakening of monsoon precipitation. However, other reasons may account for this observed weakening, such as the warming of Indian Ocean SST. We further experimented with WRF simulations, in which we conducted regional runs forced with 1980s SST and 2000s SST data with the same LULC (Supplementary Fig. S6). When WRF is forced with ERA-Interim SST, the decline in precipitation is limited to peninsular India (Supplementary Fig. S6(c)). However, when WRF is forced with CFSR SST, the area-wide decline in precipitation is quite prominent. This is possibly because the warming of WIO and SIO is not as prominent in ERA-Interim when compared to CFSR. This also agrees with findings obtained by Roxy *et al.*^11^ and Rao *et al.*^8^,^9^ that warming of SIO and WIO resulted in a decline in monsoon precipitation. The future scope of this present work is to perform detection and attribution studies for potential declines of Indian monsoons with model runs forced with SST warming only, aerosol forcing only, LULC changes only and all controlling factors together.

The limitations of the study are as follows:

1. The LULC map for 1987, as obtained from the University of Maryland (UMD), presents the dominant LULC of a selected grid^25^; hence, the final LULC map may have resulted in a small number of misclassifications due to merged classes. Some of the misclassifications may be attributed to noisy pixels, inconsistency in the ancillary data sources during training and improper choice of the metrics during classification^26^. However, the overall accuracy of the final LULC map was approximately 81%^26^, and it is widely used in various similar studies for analyzing changes in vegetation patterns and biophysical properties^27^,^28^,^29^, projecting global water resources^30^, and global data assimilation systems^31^.
2. The LULC map of 2005, as obtained from MODIS, may have misclassified some land types such as savannahs and woody savannahs, open shrub lands and grasslands due to similar biophysical and spectro-thermal properties^32^.
3. Changes in LULC might have resulted in changes in emissions of absorbing and scattering aerosols^33^. These changes would have affected the monsoon circulation, which has not been considered here.
4. The land surface model considered here uses natural hydrological processes without human interventions, such as irrigation through ground water pumping and reservoir operation, which may have significant feedback to monsoon circulation.
5. The dynamic recycling model considered here is based on the assumption of well-mixed atmospheric conditions.

Nevertheless, our findings highlight the need to consider changes in LULC in climate model projections for the Indian monsoon, because such changes have a significant role in precipitation trends. We also argue that land surface representation in climate models used for simulation of monsoon at different scales needs to be improved to consider the significant role of LULC and land surface ET on the changing behavior of the Indian monsoon.

## Methods

### Identifying Land Use Land Cover (LULC)

Here we use land use and land cover (LULC) maps for 1987 and 2005 representing land use patterns for two decades: (a) 1980–1989 and (b) 2000–2009, respectively. The 1-km UMD map is prepared based on satellite imagery collected by AVHRR from 1981 to 1994 (1987 Map). The LULC map has 14 classes and is available in three spatial scales: 1 degree (~110 km), 8 km and 1 km. We used the 1km LULC map. The classification procedure used in the map was based on red, infrared and thermal bands in conjunction with various metrics like NDVI^25^.

The MODIS land cover product is available at a 500-m spatial resolution and a 0.05° low spatial resolution for climate modeling studies. It was prepared using supervised classification and consists of maps produced for five different LULC classification schemes: a 17-class International Geosphere–Biosphere Programme classification (IGBP); a 14-class University of Maryland classification (UMD); a 10-class system used by the MODIS LAI/FPAR algorithm; an 8-biome classification; and a 12-class plant functional type. Here we used the LULC map of 500 m resolution.

In this study, both the land cover maps are resampled to 1 km. The land cover classes have been reclassified with respect to the WRF pre-defined classes for analysis (Supplementary Table 1). The land use map from 1987 does not have urban classes. In the 1987 LULC-map, snow and ice are classified as barren lands, accounting for a small fraction of land in North India over the Jammu and Kashmir regions^25^. Further, snow and ice classes are more important for winter precipitation, whereas here we focus on summer monsoon precipitation. The number of urban grids in 1987 was very small compared to the number of crop-land grids and forest grids; hence, we assume the effects of such grids will not be significant on a mesoscale precipitation pattern.

The aforementioned methodology involves steps such as resampling and reclassification along with the use of data products from multiple sources, all of which is associated with significant uncertainty and may result in deviation from the real LULC changes. However, LULC maps are not available for both periods from the same source, so we had no other options for performing this analysis. The dominant land cover in 1987, as obtained from UMD, was “wooded grassland”. However, this class does not exist in the WRF LULC schemes (nor in MODIS). The spectral properties of wooded grassland and woodland are similar; hence, we grouped them together as “woody savanna”, a classification that is consistent with WRF input LULC schemes. The other limitation of this method is the use of coarse resolution remotely sensed data for tree cover classification, which has always remained a major challenge. However, a number of studies have shown that coarse resolution satellite data can be used in characterizing land cover [De Fries *et al.*^25^; Malingreau *et al.*^34^; Loveland and Belward^35^; Xie *et al.*^36^]. Xie *et al.*^36^ enlists all the available satellite sensors and their applications and discusses the use of coarse resolution data like AVHRR and MODIS for global and continental mapping of vegetation and land cover. De Fries *et al.*^25^ developed a methodology for using AVHRR 1-km data to obtain tree-cover percentage. The Global Land Cover Characterization (GLCC) Database, generated from AVHRR 1 km data in 1992, is still widely used. In 2001, NASA released land cover products based on monthly data collected by the MODIS sensor. At a continental scale, Mayaux *et al.*^37^ mapped the vegetation cover of Central Africa using the AVHRR LAC and GAC data. These limitations lead to inconsistencies in the estimates of LULC and changes within the literature. Yamashima *et al.*^38^ showed that cropland was the dominant LULC in India during 1992. Hansen *et al.*^39^ showed minimal changes in the forestland in India from 2000 to 2012. In another study, Hansen *et al.*^26^ showed that the major land use types in India during 1992 was wooded grassland/open shrubland, using a 1-km spatial resolution data from the Advanced Very High Resolution Radiometer (AVHRR). Masson *et al.*^40^ and Myneni *et al.*^41^ have shown that the dominant land cover for India in the 1990s was wooded grassland/savannah, a finding that directly contradicts Yamashima *et al.*^38^ and agrees with our input to WRF.

### Using LULC and Vegetation Data in WRF-CLM

CLM recognizes land cover type based on a PFT classification approach where ‘the WRF input land cover (LULC) types’ are translated into the CLM4 PFTs as per Supplementary Table S2. In CLM, the surface is represented by five primary subgrid land cover types (glacier, lake, wetland, urban, vegetated) in each grid cell. The vegetated portion of a grid cell is further divided into patches of up to 4 of 16 plant functional types, each with its own leaf and stem area index and canopy height. The dominant LULC in India during 1987 was woody savannah; during 2005, it was cropland. Woody savannah consists of 80% evergreen shrub and 20% bare ground, whereas cropland is 85% crop and 15% bare ground^42^. Leaf and stem area indices (m^2^ leaf area/m^2^ ground area) are updated in CLM-WRF daily by interpolating between monthly values linearly.

The monthly values (for JJAS) of LAI as observed in MODIS image and as used in WRF through consideration of PFTs are plotted in Supplementary Fig. S7. The plots show that the PFT-based LAI has a seasonal pattern with lower values of LAI during June, which increases with the progress of a monsoon. These data are similar to those observed in MODIS. However, the PFT-based approach consistently overestimates the LAI values when compared to observations. Furthermore, the PFT-based LAI starts reducing in September, whereas the observed data show similar LAI values for both August and September. These are the limitations of the PFT-based approach, which at present is considered in the state-of-the-art Community Land Surface Model.

### Forcing Data and Model set up

Here, we used a Weather Research and Forecasting (WRF) model (version 3.6) in non-hydrostatic mode, coupled with a Community Land Surface Model (CLM) for understanding land surface feedback^44^ [Details in Supplementary Text]. The simulations were performed over the selected domain, which covers the entire Indian subcontinent with horizontal grid resolutions of 36 km (Fig. 2(a)). Lateral boundary conditions are provided by the European Centre for Medium-range Weather Forecasts (ECMWF) reanalysis data or ERA-I data^43^ with 6-hourly outputs (00, 06, 12, 18 UTC) at a 0.75° × 0.75° horizontal resolution over 30 pressure levels. We also used NCEP Climate Forecast System Reanalysis (CFSR) data^45^ at a 0.50° × 0.50° spatial resolution for two selected decades, 1979–1989 and 2000–2010, with the same model configuration. The model was run from May to October each year, and the analysis was performed for the Indian Summer Monsoon (June-July-August-September, JJAS) months, thus allowing for one month of spin-up (May) time each year. Model runs are compared with India Meteorological Department (IMD) gridded rainfall data at a 0.25° × 0.25° resolution^46^.

The consistency between the two LULC types with those used for WRF-CLM simulations is presented in Supplementary Table S1. The model simulations are performed with spectral nudging to eliminate spurious influence of the boundaries on large-scale circulation inside the regional model domain. To assess the impacts of changes in Land Use Land Cover (LULC) we performed the following simulations:

- 2000–2010 with LULC Map of 1987.
- 2000–2010 with LULC Map of 2005
- Similarly, impacts of changes in SST on monsoon rainfall, given the constant LULC, are estimated with the following simulations:
- 1979–1989 with LULC Map of 1987.
- 2000–2010 with LULC Map of 1987.

### Spectral Nudging in regional climate model

In the present study, large-scale forcing—zonal wind (U), meridional wind (V) and Temperature (T)—nudged above the Planetary Boundary Layer (PBL). Here, we use a relaxation time of 3333s (or about one hour), which would correspond to guv and gt (nudging coefficient for U and V and for T, respectively) values of 0.0003, with x-wavenumber and y-wavenumber (top wave number to nudge in x and y direction) equal to three to nudge features with wavelengths of approximately 1500 km and upward.

### Dynamic Recycling Model

We used a dynamic recycling model^22^, which is based on a Lagrangian approach, to track the moisture to compute the recycled precipitation. This model assumes a well-mixed atmospheric condition. In this study, we used an extended version of the dynamic recycling model^22^ to quantify the impact of atmospheric transport of water vapor from different evaporative sources on the Indian summer monsoon rainfall. The spatial extent of a selected domain *D,* comprising 13 different regions, are shown in supplementary Fig. S8.

The recycling ratio, or fraction of moisture evaporated from a region *1* that precipitates back to same region (i.e. region *1*), is represented by


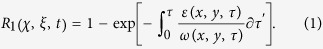


Here, *R*_*1*_ represents the fraction of moisture along the trajectory between time *t* = *τ* and *t* = 0, whereas *ε*/*ω* represents the ratio of evaporation to the precipitable water along the same trajectory. Equation 1 is used to develop the generalized expression for calculating the moisture fraction contribution from each source region along the trajectory of the water vapor.

The fraction of precipitation generated from two adjoining regions, *1* and *5*, (see Supplementary Fig. S8), can be calculated by following the trajectory of the water vapor backward in time from its current location (*x*(*t*), *y*(*t*)) to the location at the border of region *1*(*x*(*τ*_1_), *y*(*τ*_1_)), and from (*x*(*τ*_1_), *y*(*τ*_1_)) to the location in region *5*(*x*(*τ*_2_), *y*(*τ*_2_)). The evaporation over these regions (*1* and *5*) supplies moisture to the air column along the trajectory, which is used as precipitation over the sink region. Therefore, the fraction of atmospheric water present in the trajectory between the points (*x*(*t*)*, y*(*t*)) to the (*x*(*τ*_2_), *y*(*τ*_2_)) resulting from evaporation in *1* and *5* is given by


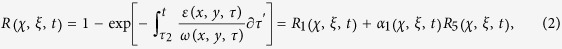


where,


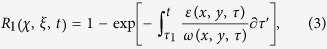



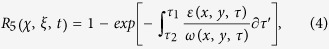


and


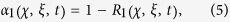


where, *R*_1_ and *R*_5_ represent the fraction of evaporated moisture collected from two regions (*1* and *5*) along the water vapor trajectory between the points(*x*(*t*), *y*(*t*))to the (*x*(*τ*_2_), *y*(*τ*_2_)). In addition, *α*_1_ represents the fraction of evaporated moisture from region 5 along the trajectory that is not lost (via precipitation) in the intermediate part of the trajectory in region 1. Similarly, the net moisture contributions from different sources along the trajectory of the water vapor can be quantified by calculating the moisture fraction corresponding to each source region.

For a domain *D* which consists of *13* different evaporative sources, the fraction of moisture collected from *N* different source regions *1*, *2*, *3, ……, 13* is represented by *R*_*1*_, *R*_*2*_, *R*_*3*_, ………, *R*_*13*_, respectively. Therefore, the total contribution at sink (*χ*, *ξ*, *t*) from all the segments within the source region ′*SR*_*k*_′, and along the trajectory of the water vapor are grouped as


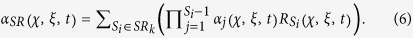


Here, *Si* represents the i^th^ segment. Similarly, the total contributions from all the segments, including those that are within the source region along the trajectory can calculated by adding contribution from all segments of the trajectory. Therefore, the fraction of moisture at the sink location (χ, *ξ*, *t*) that originated as evaporation within the domain *D* is given by


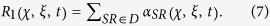


The spatially averaged contribution in terms of precipitation *P*_*r*_ from source region *SR*_*1*_ to the sink region *SN*_*1*_, for any day *t* = *d* can be calculated by


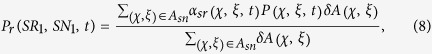


where *P*_*r*_(*SR*_1_, *SN*_1_, 1) represents the precipitation generated over ′*SN*_1_′ as a result of evaporation ′*SR*_1_′. Here, *P*(*χ*, *ξ*, *t*) represents the total precipitation over the point (*χ*, *ξ*) for *‘t’* day, and *δA*(*χ*, *ξ*) represents the area of each grid cell.

Regions selected for this study are shown in Supplementary Fig. S8.

## Additional Information

**How to cite this article**: Paul, S. *et al.* Weakening of Indian Summer Monsoon Rainfall due to Changes in Land Use Land Cover. *Sci. Rep.* **6**, 32177; doi: 10.1038/srep32177 (2016).

## Supplementary Material

Supplementary Information
