# Supplementary material for: Weakening of Indian Summer Monsoon Rainfall due to Changes in Land Use Land Cover
Source: Sci Rep. 2016 Aug 24;6:32177. doi: 10.1038/srep32177 (PMC4995379; doi:10.1038/srep32177)
Supplement: Supplementary Information [file srep32177-s1.doc]

**Supplementary Information for:**

**Weakening of Indian Summer Monsoon Rainfall due to Changes in Land Use Land Cover**

Supantha Paul^1^, Subimal Ghosh^1,2*^, Robert Oglesby^3,4^, Amey Pathak^2^, Anita Chandrasekharan^2^, RAAJ Ramshankaran^1,2^

^1^Interdisciplinary Program in Climate Studies, Indian Institute of Technology Bombay, Mumbai- 400 076, India

^2^Department of Civil Engineering, Indian Institute of Technology Bombay, Mumbai- 400076, India

^3^ Department of Earth & Atmospheric Sciences, University of Nebraska, Lincoln, NE, USA

^4^ School of Natural Resources, University of Nebraska, Lincoln, NE, USA

^*^Corresponding Author, Email: [subimal@civil.iitb.ac.in](mailto:subimal@civil.iitb.ac.in) , Phone: +91 22 2576 7319

# Supplementary Text

# Weather Research and Forecasting (WRF) Model

The Weather Research and Forecasting (WRF) Model is a mesoscale numerical weather prediction system designed to serve both atmospheric research and operational forecasting^S1^. The equation set for Advanced Research Weather forecasting (ARW) is fully compressible, Eulerian and non-hydrostatic with a run-time hydrostatic option. It is conservative for scalar variables. The model uses terrain-following, hydrostatic-pressure vertical coordinate with the top of the model being a constant pressure surface. The horizontal grid is the Arakawa-C grid. The time integration scheme in the model uses the third-order Runge-Kutta scheme and the spatial discretization employs 2^nd^ to 6^th^ order schemes^S1^.

WRF includes several physical parameterization schemes for modelling earth’s atmosphere and associated different land surface feedback processes. In this study, cloud microphysical processes are modelled using WSM 5-class scheme^S2^ which predicts hydrometeors like water vapor, cloud vapor, cloud ice, rain and snow. Existence of super-cooled water and gradual melting of snow falling below the melting layer are considered here. Sub-grid scale cloud processes are parametrized using new Kain–Fritsch cumulus parameterization scheme^S3^. The KF-scheme includes both shallow and deep convection, a convective trigger function and a closure assumption. Convective updrafts are represented by using a steady-state entraining–detraining plume model. Planetary boundary layer (PBL) is being modelled with YSU scheme^S4^. This scheme uses a non-local first order closure which has more accuracy for simulating deeper vertical mixing in PBL and has an explicit treatment of entrainment at the top of the PBL. Rapid Radiative Transfer Model (RRTM)^S5^ scheme is used for long wave radiation. RRTM considers multiple bands and trace gases in the atmosphere. Sub-division of spectral bands helps to calculate contributions from the major absorbing species (e.g. water vapor, CO_2_, O_3_, CH_4_, N_2_O, CFC-11, CFC-12, and CFC-22) with more accuracy alongside radiative transfer. The short wave radiation scheme is based on Dudhia (1989)^S6^. This scheme takes into account downward integration of solar flux related to clear-air scattering, water vapor absorption, and cloud absorption and reflection. Momentum and heat transfer from the ground is modeled by the Monin-Obukhov surface layer scheme^S7-S10^. Land surface is modelled using Community Land Surface Model^31^. A list of the schemes is provided in Supplementary Table 3.

The regional model receives information through its boundary which can come from GCM’s or reanalysis dataset. This interaction with the boundary forcing may lead to the alteration of the large scale circulations. This problem is due to the over-specification of boundary conditions for the atmospheric equations that are solved in a grid^S11^. Rinke and Dethloff^S12^ found that most of the contribution to the error in the regional model comes from deviations in the large scales. The errors in the synoptic circulation affects precipitation and other variables inside regional model domain. Miguez-Macho et al.^S13^ reported that the error in the location of the main precipitation pattern is largely due to a systematic distortion of the large-scale flow by the interaction with the lateral boundaries, and not to physical parameterizations or the initialization of soil moisture. Large scales perturbations are thus, functions of domain geometry, as well as the topography in the interior of the grid. Spectral nudging of the long waves is therefore, essential for accurate simulation of small scale circulation and to eliminate spurious influence of the boundaries on large scale circulation inside the regional model domain^S13^. It keeps the simulated state close to the driving state at larger scales, while generating smaller-scale features at regional scale.

# Community Land Surface model (CLM4.0)

Community land surface model (CLM4.0) is an outcome of continuous development of land surface schemes used for NCAR GCMs. Land surface is divided into three principal subgrid components, viz. landunits, columns and Plant Functional Types (PFTs) arranged hierarchically. The landunit is further divided into glacier, lake, wetland, urban, and vegetated as percentage of each grid cell. At column level, primarily snow and soil variability are captured. Each landunit can have multiple columns with defined state variables and their respective fluxes for water and energy. Additionally, soil column consists of fifteen layers of soil whereas there are five layers for snow. At this column level, boundary fluxes are calculated as weighted average over PFT’s. At PFT level, sub-grid scale heterogeneity is prescribed through fractional allocation of land cover to four or more Plant Functional Types (PFTs)^S14,S15^. The composition and abundance of PFTs within a grid cell is prescribed as time-invariant fields. At this level, surface fluxes and vegetation state variables are defined alongside the leaf area index and the fraction of canopy as physical state variables.

Some of the improvements that are incorporated in CLM4 include modelling soil moisture, albedo, urban heat island effect and dynamic land cover change^44^. In this study CLM4 was coupled with WRF3.6. As oppose to Noah, CLM model soil moisture below tenth soil layer of the ground, subsequently demanding more computational resources.

# Supplementary Reference

1. Skamarock W. C. et al. A description of the advanced research WRF version 3. *NCAR Technical Note*, NCAR, Boulder, Colo, USA (2008).
2. Hong, S-Y., Dudhia J. & Chen S–H. A revised approach to ice microphysical processes for the bulk parameterization of clouds and precipitation. *Mon Wea Rev* **132,** 103–120(2004).
3. Kain J. S. The Kain–Fritsch convective parameterization: An update. *J Appl Meteor* **43,** 170–181(2004).
4. Hong, S–Y., Noh, Y. & Dudhia, J. A new vertical diffusion package with an explicit treatment of entrainment processes. *Mon Wea Rev* **134,** 2318–2341(2006).
5. Mlawer, E. J., Taubman, S. J., Brown, P. D., Iacono, M. J. & Clough, S. A. Radiative transfer for inhomogeneous atmospheres: RRTM, a validated correlated–k model for the longwave. *J Geophys Res* **102,** 16663–16682(1997).
6. Dudhia J. Numerical study of convection observed during the Winter Monsoon Experiment using a mesoscale two–dimensional model. *J Atmos Sci* **46,** 3077–3107(1989).
7. Paulson, C. A. The mathematical representation of wind speed and temperature profiles in the unstable atmospheric surface layer. *J Appl Meteor* **9,** 857–861(1970).
8. Dyer, A. J. & Hicks, B. B. Flux–gradient relationships in the constant flux layer. *Quart J Roy Meteor Soc* **96,** 715–721(1970).
9. Webb, E. K. Profile relationships: The log-linear range, and extension to strong stability. *Quart J Roy Meteor Soc* **96**, 67–90(1970).
10. Beljaars, A.C.M. The parameterization of surface fluxes in large-scale models under free convection. *Quart J Roy Meteor Soc* **121**, 255–270(1994).
11. Staniforth, A. Regional modeling: A theoretical discussion. *Meteorol Atmos Phys* **63,** 15–29(1997).
12. Rinke, A. & Dethloff, K. On the sensitivity of a regional Arctic climate model to initial and boundary conditions. *Clim Res* **14(2),** 101–113(2000).
13. Miguez-Macho, G., Stenchikov, G. L. & Robock, A. Spectral nudging to eliminate the effects of domain position and geometry in regional climate model simulations. *J Geophys Res* **109,** D13104, doi:10.1029/2003JD004495 (2004).
14. Bonan, G.B. et al. The land surface climatology of the Community Land Model coupled to the NCAR Community Climate Model. *J Clim* **15,** 3123-3149(2002a).
15. Bonan, G.B., Levis, S., Kergoat, L. & Oleson, K.W. Landscapes as patches of plant functional types: An integrating concept for climate and ecosystem models. *Glob Biogeochem Cycles* **16,** 5.1-5.23(2002b).

S16. Knyazikhin ,Y., et al. MODIS Leaf Area Index (LAI) and Fraction of Photosynthetically Active Radiation Absorbed by Vegetation (FPAR) Product (MOD15) Algorithm Theoretical Basis Document, http://eospso.gsfc.nasa.gov/atbd/modistables.html(1999).

S17. Lawrence, P. J., & Chase T. N. Representing a new MODIS consistent land surface in the Community Land Model (CLM 3.0), J. Geophys. Res., 112, G01023, doi:10.1029/2006JG000168 (2007).

# Supplementary Figures


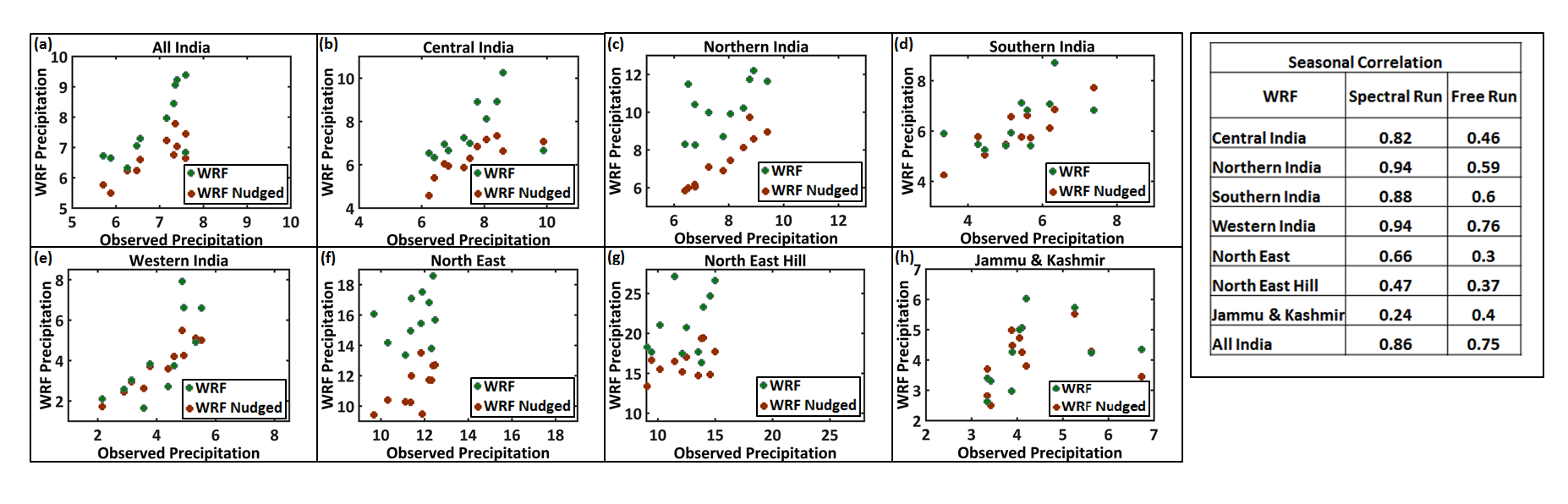


**Supplementary Figure S1.** Evaluation of WRF simulations of seasonal precipitation for free and with spectrally nudged runs. The scatter plots are for (a) All India, (b)-(h) and its meteorologically homogeneous regions. The table shows the seasonal correlations and improvements with nudged runs. Maps are prepared with MATLAB R2012b (http://in.mathworks.com/products/new_products/release2012b.html).


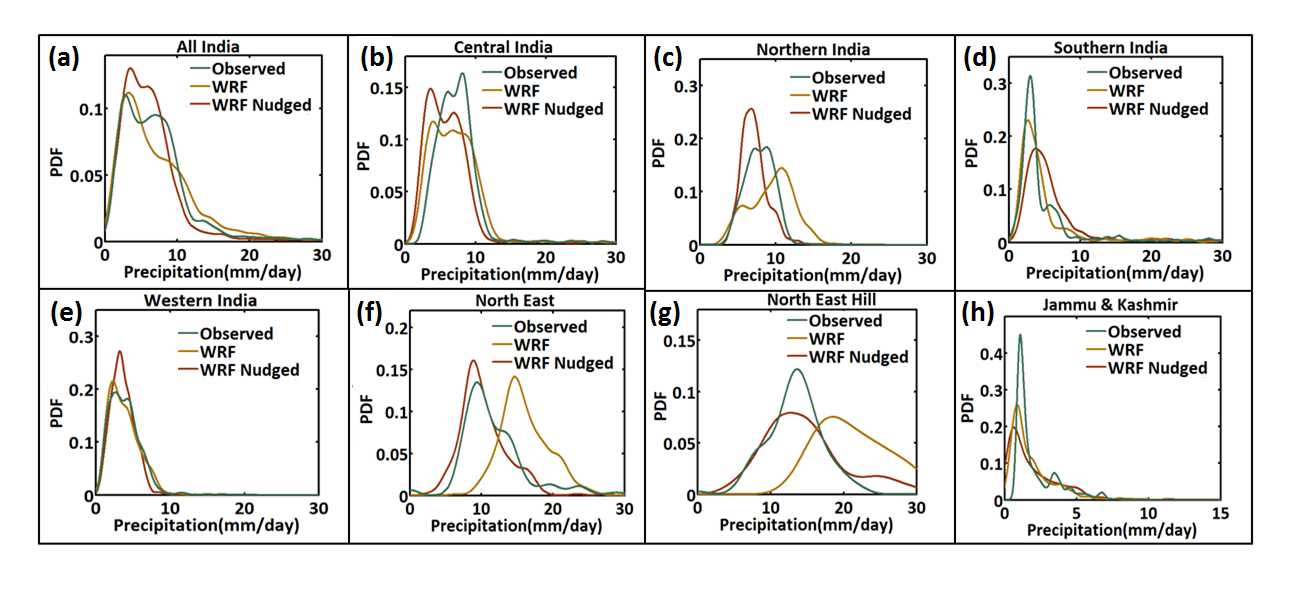


**Supplementary Figure S2.** PDFs representing the spatial distribution monsoon precipitation for observed, free simulations and spectrally nudged simulations. (a) All India, (b)-(h) zone wise. Maps are prepared with MATLAB R2012b (http://in.mathworks.com/products/new_products/release2012b.html).


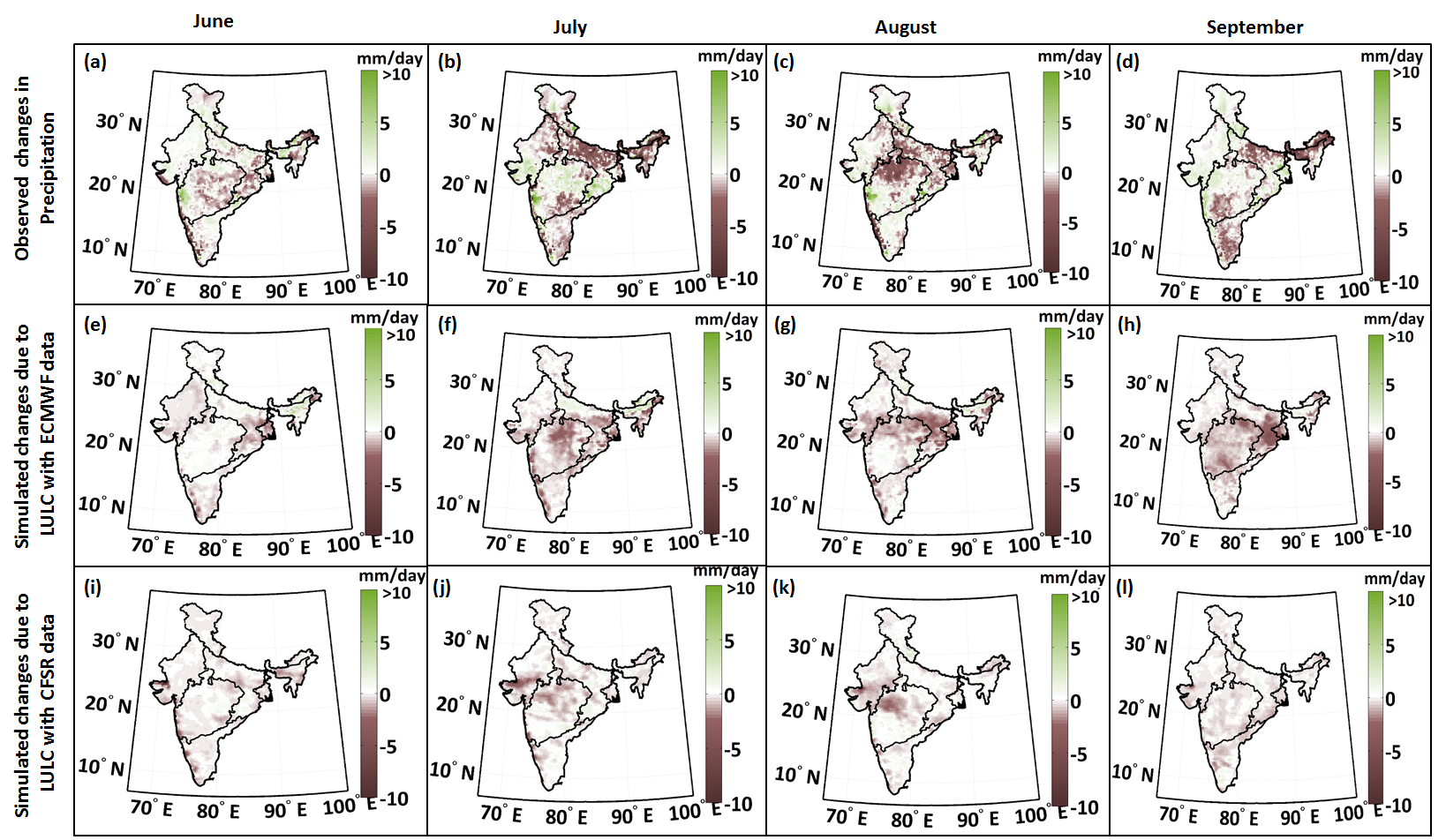


**Supplementary Figure S3.** (a)-(d) observed changes in precipitation during June, July August and September, respectively., (e)-(h) monthly precipitation differences between the simulations with different LULC, using ECMWF reanalysis data, (i)-(l) and CFSR data. Maps are prepared with MATLAB R2012b (http://in.mathworks.com/products/new_products/release2012b.html).


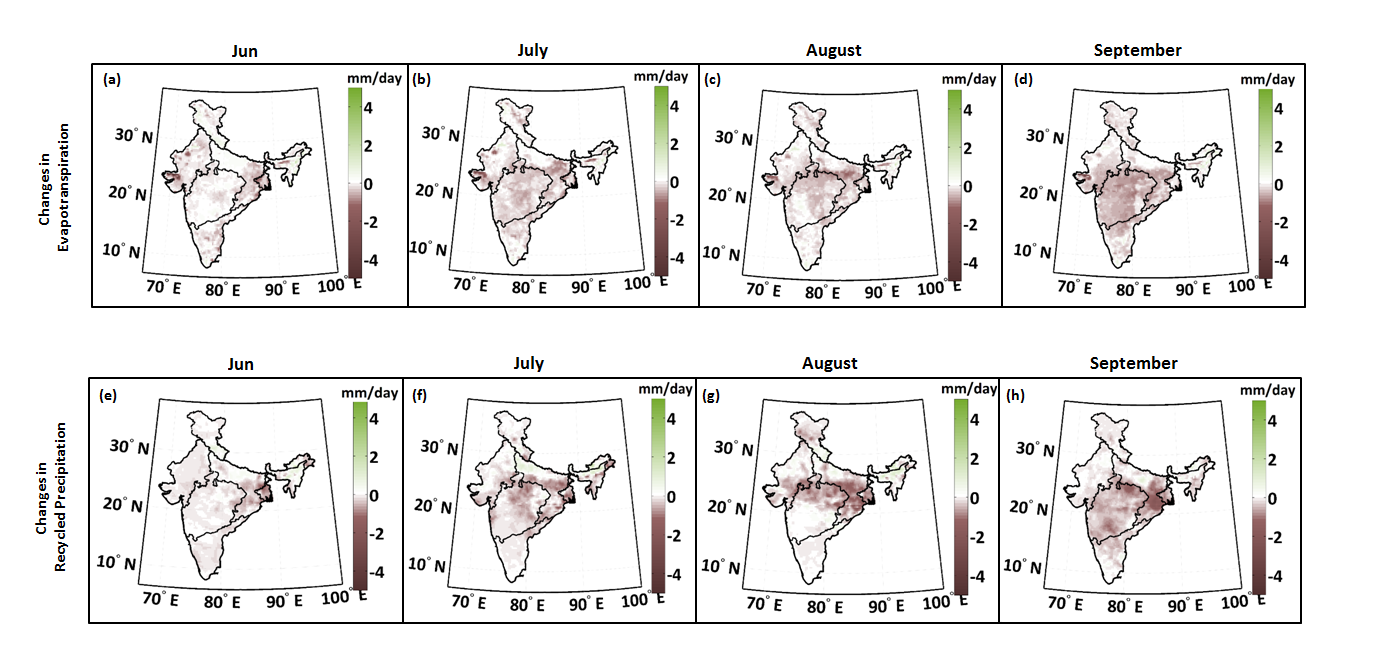


**Supplementary Figure S4.** Monthly differences between simulated ET ((a)-(d)), and recycled precipitation ((e)-(h)) over JJAS when WRF is simulated with different LULC. Maps are prepared with MATLAB R2012b (<http://in.mathworks.com/products/new_products/release2012b.html>).


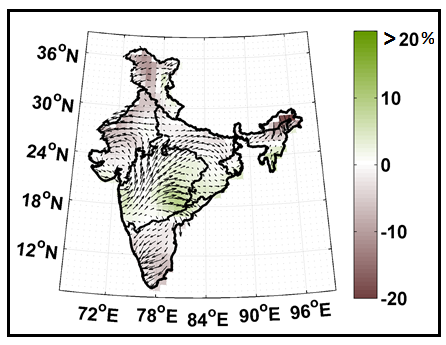


**Supplementary figure S5.** Observed Changes in wind between historical (1979-1989) and present decade (2000-2010) as obtained from ECMWF data. Maps are prepared with MATLAB R2012b (http://in.mathworks.com/products/new_products/release2012b.html).


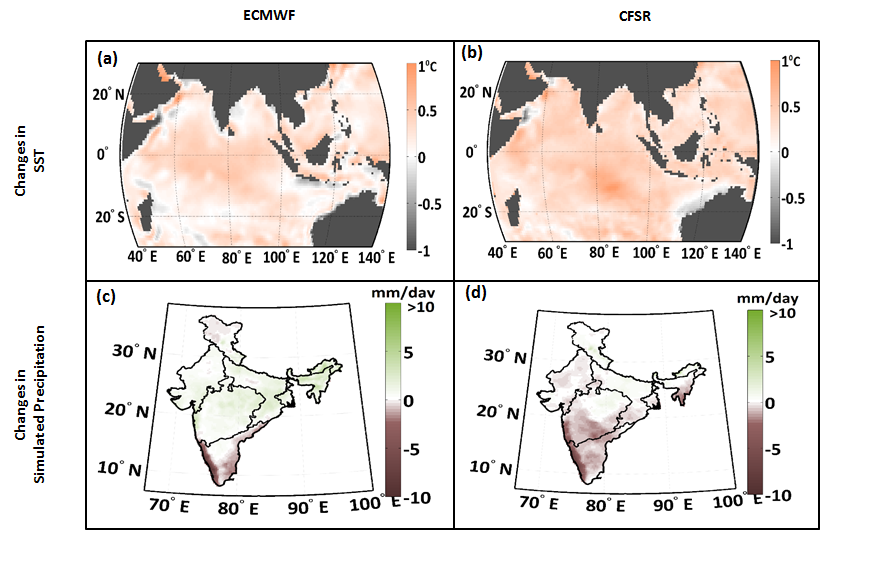


**Supplementary Figure S6.** (a)-(b) Differences in SST between the decades 1979-89 and 2000-10 with ERA-Interim (a) and CFSR (b) data and their simulated impacts on monsoon rainfall ((c)-(d)). Maps are prepared with MATLAB R2012b (http://in.mathworks.com/products/new_products/release2012b.html).


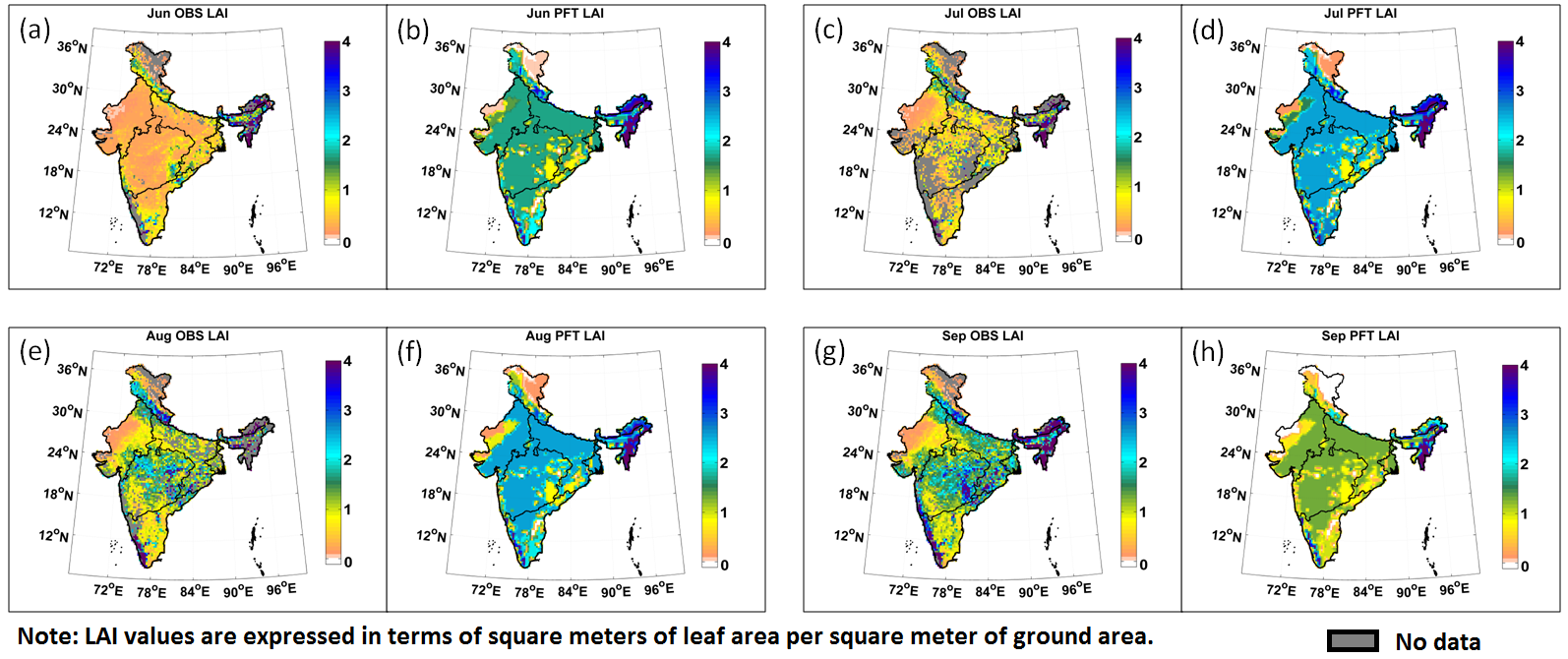


**Supplementary figure S7**. Comparison of leaf area index (LAI), CLM PFT-LAI against MODIS observed LAI^S16^ following Lawrence & Chase^S17^. Maps are prepared with MATLAB R2012b (http://in.mathworks.com/products/new_products/release2012b.html).


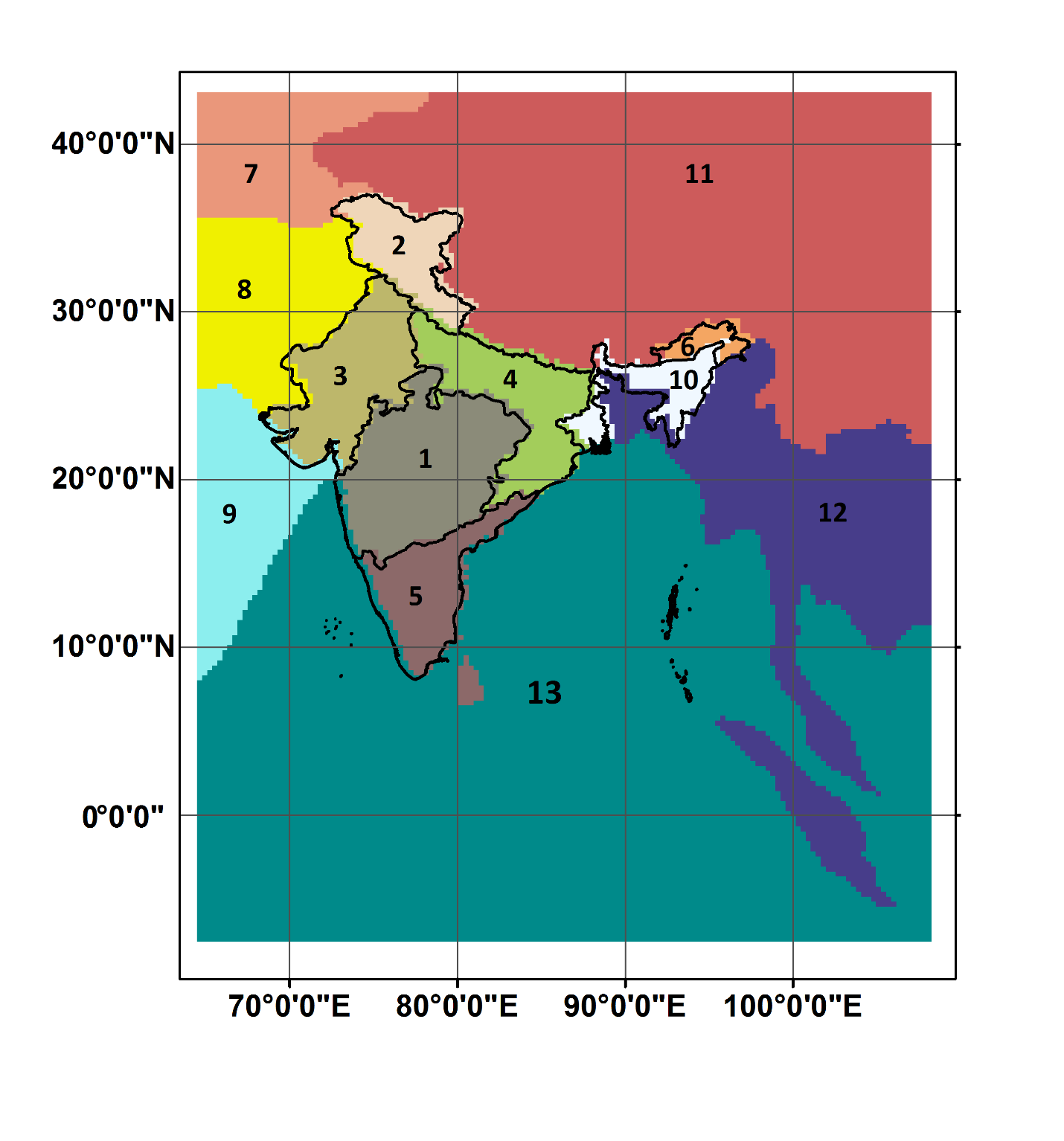


**Supplementary figure S8.** Regions used for Dynamic Recycling Model. Maps are prepared with ArcGIS10.0 (http://www.esri.com/software/arcgis/arcgis-for-desktop).

# Supplementary Tables

**Supplementary Table S1:** Land use Classes Used for the study

| **UMD classification scheme** | **Reclassified UMD classes** | **IGBP**  **classification**  **scheme** | **Landuse classes Used in the WRF** |
| --- | --- | --- | --- |
| Evergreen Needleleaf | Evergreen Needleleaf | Evergreen Needleleaf | Evergreen Needleleaf Forest |
| Evergreen Broadleaf | Evergreen Broadleaf | Evergreen Broadleaf | Evergreen Broadleaf Forest |
| Deciduous Needleleaf | Deciduous Needleleaf | Deciduous Needleleaf | Deciduous Needleleaf Forest |
| Deciduous Broadleaf | Deciduous Broadleaf | Deciduous Broadleaf | Deciduous Broadleaf Forest |
| Mixed Forest | Mixed Forest | Mixed Forest | Mixed Forests |
| Closed Shurbland | Closed Shurbland | Closed Shrubs | Closed Shrublands |
| Open Shrubland | Open Shrubland | Open Shurbs | Shrubland |
| Woodland | Woody Savannahs | Woody Savannahs | Woody Savannahs |
| Wooded Grassland |
|  |  | Savannahs | Savannahs |
| Grassland | Grasslands | Grasslands | Grasslands |
|  |  | Permanent Wetlands | Permanent Wetland |
| Cropland | Croplands | Croplands | Cropland/Agriculture |
|  |  | Urban and Built Up | Urban and Built-Up |
|  |  | Croplands and Natural Vegetation Mosaics | Cropland/natural vegetation mosaic |
|  |  | Snow and Ice | Snow and Ice |
| Bare Ground | Bare Ground | Barren and Sparsely Vegetated | Barren or Sparsely Vegetated |
| Water | Water | Water | Water |

**Supplementary Table S2** Mapping of input LULC type into CLM Plant functional type(PFT)S15

| **Distribution of Plant Functional Type** | | |  | | | | | |
| --- | --- | --- | --- | --- | --- | --- | --- | --- |
| **Class**  **No.** | **Landuse Land Cover  Type**  **(LULC)** | **Patch 1** | | | **Patch 2** | | **Patch 3** | |
|  |  | PFT | | Cover | PFT | Cover | PFT | Cover |
| 1 | Evergreen needleleaf forest | NET | | 0.75 | B | 0.25 | - | - |
| 2 | Evergreen broadleaf forest | BET | | 0.95 | B | 0.05 | - | - |
| 3 | Deciduous broadleaf forest | BDT | | 0.75 | B | 0.25 | - | - |
| 4 | Mixed forest | NET | | 0.37 | BDT | 0.37 | B | 0.26 |
| 5 | Closed shrub | ES | | 0.80 | B | 0.20 | - | - |
| 6 | Open Shrub | ES | | 0.50 | WG | 0.30 | B | 0.20 |
| 7 | Woody Savanna | ES | | 0.80 | B | 0.20 | - | - |
| 8 | Savannah | WG | | 0.70 | TST | 0.30 | - | - |
| 9 | Grassland | WG | | 0.60 | CG | 0.20 | B | 0.20 |
| 10 | Permanent Wetland | B | | 1.0 | - | - | - | - |
| 11 | Cropland | C | | 0.85 | B | 0.15 | - | - |
| 12 | Urban and Built-Up | B | | 1.0 | - | - | - | - |
| 13 | Cropland/natural vegetation mosaic | C | | 0.50 | WG | 0.35 | B | 0.15 |
| 14 | Snow and Ice | B | | 1.0 | - | - | - | - |
| 15 | Barren or Sparsely Vegetated |  | |  |  |  |  |  |
| 16 | Water | B | | 1.0 | - | - | - | - |

**N.B.** NET, needleleaf evergreen tree; BET, broadleaf evergreen tree; BDT, broadleaf deciduous tree; TST, tropical seasonal tree; ES, evergreen shrub; DS, deciduous shrub; ADS, arctic deciduous shrub; CG, C3 grass; WG, C4 grass; AG, arctic grass; C, crop; B, bare.

**Supplementary Table S3** Physical parameterization schemes used for the study

| **Physics** | **Schemes used for this study** |
| --- | --- |
| Cloud Micro physics | WSM 5-class scheme^S2^ |
| Sub grid scale cloud | Kain-Fritsch (new Eta) scheme^S3^ |
| Planetary Boundary-layer(PBL) | YSU scheme^S4^ |
| Long wave radiation | rrtm scheme^S5^ |
| Short wave radiation | Dudhia scheme^S6^ |
| Surface-layer physics | Revised MM5 Monin-Obukhov scheme^S7-S10^ |
| Land-surface physics | Community Land surface model^31^ |
